# Clitoral reconstruction and psychosexual care after female genital mutilation/cutting: Assessment of multidisciplinary care

**DOI:** 10.1177/17455057251315814

**Published:** 2025-03-31

**Authors:** Muriel Meyer, Gideon Sartorius, Jasmine Abdulcadir

**Affiliations:** 1 University of Basel, Basel, Switzerland; 2 Women’s Clinic, Basel University Hospital, Basel, Switzerland; 3 Department of Pediatrics, Obstetrics and Gynecology, Geneva University Hospitals (HUG), Genève, Switzerland

**Keywords:** clitoral reconstruction, female genital mutilation, female genital cutting, multidisciplinary care, psychosexual care

## Abstract

**Background:**

Multidisciplinary care following female genital mutilation/cutting (FGM/C) can consist of surgical interventions such as clitoral reconstruction (CR) in combination with individualized psychosexual care (PC). Evidence on both treatments, alone or in association, is limited.

**Objectives:**

To investigate the overall satisfaction with PC only or PC in combination with CR.

**Design:**

Cross-sectional study of women who attended the FGM/C outpatient clinic under study and asked for CR between January 2013 and November 2021.

**Methods:**

Data were collected through questionnaire-based interviews on motivations for asking for CR, psychological well-being (World Health Organization-Five Well-Being Index), sexual response (Female Sexual Function Index), vulvar pain, genital self-image (Female Genital Self-Image Scale), overall satisfaction with the care received, and, in the case of CR, postoperative complications.

**Results:**

The 20 women included underwent therapy primarily to feel repaired or reconstructed and to improve their sexual function. Mean overall satisfaction with the care was rated 8.95/10 ± 1.39. Twelve (60%) received CR in combination with PC. Eight (40%) received PC only. Women who received combined therapy reported higher overall satisfaction (9.17 versus 8.57), psychological well-being (17.8 versus 9.25), sexual response (31.22 versus 21.56), and genital self-image (25.60 versus 17.60) in comparison with those who only received PC. The main surgical complications were minor postoperative bleeding and one painful wound infection triggering a relapse of posttraumatic stress disorder.

**Conclusion:**

Tailored PC and CR after FGM/C seem to improve psychosexual health, well-being, body image, and pain.

**Registration:** ClinicalTrials.gov (NCT05026814)

## Introduction

According to the World Health Organization (WHO), female genital mutilation or cutting (FGM/C) includes “all procedures that involve partial or total removal of the external female genital organs, or other injuries to the female genitals for non-medical reasons.” The WHO has adopted the term *female genital mutilation* (FGM) and classifies four distinct types described in Table 1.^1^ Other scholars have increasingly proposed broadly discussing (non-)consensual and (non-)therapeutic genital modifications, regardless of the person’s gender and sex.^2–4^ Fusaschi, therefore, proposed the term “gendered genital modifications.”^3^ In this paper, we use the term FGM/C while acknowledging that the terminologies are evolving.

**Table 1.** FGM subtypes according to WHO.

Type I: Partial or total removal of clitoral glans and/or prepuce	Ia: Removal of clitoral hood or prepuce only Ib: Removal of clitoral glans with prepuce
Type II: Partial or total removal of clitoral glans and labia minora, with or without excision of labia majora (excision)	IIa: Removal of labia minora only IIb: Partial or total removal of clitoral glans and labia minora IIc: Partial or total removal of clitoral glans, labia minora, and labia majora
Type III: Narrowing of vaginal opening with creation of a covering seal by cutting and repositioning labia, with or without excision of clitoral prepuce and glans (infibulation)	IIIa: Removal and apposition of labia minora IIIb: Removal and apposition of labia majora
Type IV: Other harmful procedures	All other harmful procedures to female genitalia for nonmedical purposes, for example, pricking, piercing, incising, scraping, and cauterization of the genital area

FGM: female genital mutilation; WHO: World Health Organization.

FGM/C is often practiced during childhood in African, Middle Eastern, and some Asian countries.^5^ Due to migration flows, women and girls with FGM/C also live in countries where this practice was not originally prevalent. An estimated 24,600 women and girls with or at risk of FGM/C live in Switzerland,^6^ 600,000 in Europe,^7^ and 230 million worldwide.^8^ In some areas and among some ethnic groups, the practice seems to be decreasing due to the introduction of laws, changes in social norms, education, and increased awareness,^9^ while in others, it remains stable.^8^

FGM/C can significantly affect individuals physically and mentally over their lifetime, in the immediate, short, and long term.^10^ The impact of FGM/C on well-being can widely vary due to factors such as the type of FGM/C (Table 1), preexisting health conditions of a psychological or physical nature, the setting and modalities of the procedure, age and experience at the time of the practice, complications, quality of life, body and genital self-perception, relationships, additional traumatic experiences, and the availability of information and care concerning the possible complications of FGM/C.^11–14^ Gynecologists and other health professionals should be able to offer trained, evidence-based, tailored, trauma-informed, and socioculturally sensitive care, including surgery when indicated. They should avoid pathologizing or stigmatizing any patient, especially in the absence of symptoms.^11,15^ They should also avoid neglecting the negative consequences or suffering linked to FGM/C, even in the absence of a severe or visible scar.^16^

Current guidelines and expert opinions on the care of women and girls with FGM/C suggest that clitoral reconstructive surgery (CR) after FGM/C should be associated with psychosexual care (PC) and offered as part of multidisciplinary care.^15,17–23^ Most CR and PC are available in European countries, and the costs are fully or partially covered by the healthcare system. In France and Germany, CR is available and covered by the social security system.^24^ In Switzerland,^25^ Belgium, and the Nordic countries, PC and CR are also covered by health insurance, but sometimes only if performed by a special unit within the public health system. Only recently has training on CR and the accompanying care been offered for surgeons, particularly in countries where the prevalence of FGM/C is high,^26,27^ and the first international masterclass on the topic was only organized in 2024.^28^

In the United States and African countries, CR is generally only available as a self-pay procedure,^24^ and many women do not have the financial means to pay for it.^29^

In the centers offering multidisciplinary care in Europe, patients are usually seen by an interdisciplinary team of surgical gynecologists and sometimes plastic surgeons, sexologists, psychologists, psychiatrists, who are often trained in trauma therapy, and midwives.^17–20,23^ The importance of relational and psychotraumatic care has also been emphasized.^23^

CR is requested by and conducted on patients for different reasons, such as the hope of improving clitoral sensation, reducing or treating spontaneous or provoked clitoral pain, and improving genital self-image and female identity.^30^ Jordal et al. described five interconnected key factors for undergoing CR among women: (1) “symbolic restitution”—undoing the harm of FGM/C; (2) repairing visible stigma; (3) improving sex and intimacy through physical, aesthetic, and symbolic recovery; (4) eliminating physical pain; and (5) personal project giving hope.^31^ Villani noted similar motivations in her qualitative study, emphasizing that sociocultural influences have the power to define women solely by their alleged mutilation, with the denigrated physical change becoming a marker of their identity. The influencing sociocultural factors—such as racial hierarchy, the stigmatization of FGM/C, opportunities for self-transformation, and sociocultural notions of sexuality—may result in a desire to “escape the stigma” of FGM/C and to feel like a “complete woman.”^32^

The literature has described different techniques and modifications for clitoral reconstruction (CR). They are summarized in Table 2, which has been modified and updated according to a review coauthored by one of the authors.^22^ There are also other updates of the existing techniques that have been presented at conferences but have not been published. Additional techniques with flaps have been described for reconstructing the labia.^33^ Grafts and regenerative medicine have been studied as a method for accelerating clitoral healing or improving the aesthetic and functional result.^34–36^ The Foldès/Thabet CR technique is currently the most used and reproducible approach. It consists in removing the periclitoral scar, sectioning the suspensory ligament, and reexposing or elevating the remaining clitoral body to make it more visible and more exposed to stimuli.^20^

**Table 2.** Surgical techniques for CR.

Described by	Description of surgical technique
Narjani^37^ and Bonaparte^38^	Section of suspensory ligament of clitoris allowing repositioning of clitoral glans closer to vagina (described not in women with FGM/C but as technique for facilitating orgasm through vaginal penetration).
Thabet and Thabet^39^ and Foldès et al.^40^	Incision of cutaneous scar tissue in area of palpable clitoris with subsequent dissection of clitoral body. Currently, such cutaneous incisions can have different shapes depending on surgeon (lozenge, inversed *V*, omega, linear vertical). Section of suspensory ligament. Fixation of “neoclitoris” near vaginal entrance. Neoclitoral shaft fixed to bulbocavernosus muscles.
Ouédraogo et al.^41^	Modification of Foldès/Thabet technique without fixation stitch between clitoris and bulbocavernosus muscles.
O’Dey^33^	Reconstruction of vulva with aOAP flap, OD flap for prepuce, and microsurgical procedure called neurotizing and molding of clitoral body, mobilized after sectioning suspensory ligament (NMCS procedure).
Chang et al.^42^ and Christopher et al.^43^	Sharp circular dissection of scar tissue around the clitoral body without scar removal. Dissection of residual clitoris up to os pubis without section of suspensory ligament. Exposure of clitoris by rolling and fixation of labia majora to periosteum using sutures. Re-epithelialization under nonstick dressing. Buccal mucosa graft on raw surfaces of clitoris. Eventual fat grafting on mons pubis and labia.
Mañero and Labanca^44^	Modification of Foldès/Thabet technique with mucosal graft from posterior vaginal wall. Graft is placed over clitoris and can also be used for reconstructing inner labia if needed/desired. Neoclitoral shaft fixed to periosteum.
Wilson and Zaki^45^	CR according to Foldès and covering with labial flaps donated from remains of inner labia.
Botter et al.^20^	Modification of the Foldès/Thabet technique with a small, inverted *V*-shaped incision. No skin is resected, as this can serve as reconstruction of inner labia.
Tognazzo et al.^35^	Foldès/Thabet technique with administration of A-PRP into bodies of clitoris to accelerate healing and re-epithelialization.

aOAP: anterior obturator artery perforator flap; OD flap: omega-domed flap; NMCS: Neurotising and molding of the clitoral stump; FGM/C: female genital mutilation/cutting; CR: clitoral reconstruction; A-PRP: autologous platelet-rich plasma.

The evidence regarding multidisciplinary care, which consists of PC with or without CR, is still limited. There are only a few single-center studies that have assessed the nonsurgical care or PC received by women who requested CR.^18,46^ Most of the studies on sexual function, vulvar pain, and postoperative complications after CR have focused on different outcome measures and have used different evaluation tools, impeding interstudy comparability.^47,48^ Furthermore, previous studies have exhibited methodological limitations such as retrospective study designs,^41,44,49^ small population numbers,^44^ missing data at follow-up (up to 71%),^40^ and heterogeneity in surgical techniques. Auricchio et al. concluded that the existing evidence must be interpreted with caution, yet CR appears to have a positive impact on sexual pleasure and function, self-image, pain, and dyspareunia.^30^ Berg et al. showed self-reported reduction in sexual pain and improvement in sexual desire, arousal, orgasmic capacity, and sexual pleasure for most women. Nevertheless, up to 22% of women reported a worsening in sexuality-related outcomes (decrease in orgasms, desire, frequency of sex, and pleasure) after CR.^48^

In 2020, the first ethical analysis regarding CR insisted on the importance of patient autonomy and information. The authors concluded that CR may be performed for clitoral pain and associated sexual dysfunction, as well as when a patient sees the surgery as a form of physical and psychological repair of FGM/C despite the absence of pain or other physical or psychosexual symptoms. In such cases, they discussed the importance of providing adequate education regarding common misconceptions, the anatomy and function of the vulva, sexual response, the evidence for CR, and the best possible culturally informed psychosexual therapy.^22^

Experts agree that not all women who experience FGM/C need or request CR and that CR plays an important symbolic role beyond improving anatomical and clitoral function.^20^ The WHO recommends psychological support, health education, information about FGM/C, and offering sexual counseling and care. Because of the lack of high-quality data in 2016 and 2018, the WHO could not make a conclusive recommendation in favor of or against CR based on evidence.^50^ The updated guidelines will certainly include new evidence^51^ published since then.

Our questionnaire-based cross-sectional study aimed to assess patients’ satisfaction with PC when it was provided with or without CR.

## Material and methods

### Eligibility, recruitment, and ethical considerations

Our questionnaire-based cross-sectional study was conducted between November 1, 2021 and the end of March 2022 in a specialized clinic founded in 2010. All the women who visited the clinic under investigation between January 1, 2013 and 30 November 2021, and were actively seeking CR at their first appointment were contacted to participate in an interview to be enrolled in the study. They were informed about the study and of the possibility of withdrawing from it at any time. Women who were at least 18 years old and could read and speak the study languages (French or English) were eligible for enrollment.

**Figure 1.**
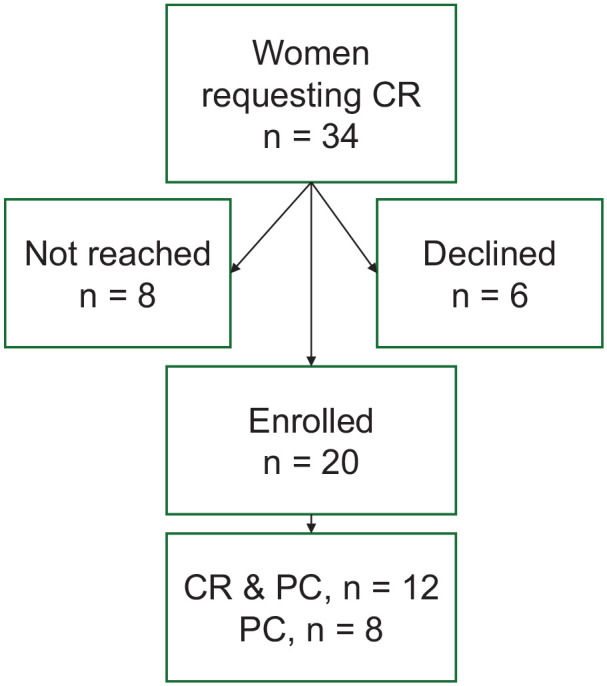
Patient enrollment.

Out of 34 women contacted by phone or letter, 20 were included in the study after signing an informed consent document. The data from the women who declined to participate or could not be contacted were not analyzed. The patient enrollment is described in Figure 1.

### Data collection

Data were collected at least 3 months post-treatment through questionnaire-based interviews (MM) and reviews of the participants’ medical records (MM and JA). Three months after surgery is normally the time when patients can resume genital sex and masturbation as the clitoris is re-epithelialized and the postoperative pain has usually subsided. The participants’ medical records were reviewed to analyze any postoperative complications recorded by the surgeon. Questionnaire-based interviews were used to assess the participants’ general satisfaction, sexual function, vulvar pain, and genital self-image. MM conducted the interviews after having been trained by JA and having shadowed JA’s clinic consultations.

The interview consisted of questions (the questionnaire can be found in the Supplemental Appendix) on:

- Sociodemographic information;
- Motivations for asking for CR with predetermined response options based on the literature^52^;
- Presurgery expectations and reasons for deciding against or in favor of CR (these were collected in plain text and analyzed using keywords to create categories using an adapted cluster analysis);
- General satisfaction with treatment.

The interview also included the following validated questionnaires:

- The Female Sexual Function Index (FSFI),^43,49,53,54^ which has been validated in French and English;
- The Female Genital Self-Image Scale (FGSIS), ^55^ which has been validated in English;
- WHO-Five Well-Being Index (WHO-5).^56^

Due to the retrospective study design, comparisons to before undergoing care cannot be reliably assessed.

The FSFI and FGSIS have already been used in previous studies on sexual function, defibulation, and CR in women with FGM/C.^44,53^

Although the FSFI, the FGSIS, and the WHO-5 are validated tools at least in English,^55–58^ they have not been validated in a specific population of women with FGM/C, which is probably due to the fact that the population of women with FGM/C is very diverse with differing countries of origin, cultural backgrounds, religions, and languages. Validating questionnaires in such a diverse population might be difficult.

The interviews were held face to face, in person, via online meetings, or by phone, according to the preference of the participant.

The study questionnaires were administered using the secured web platform REDCap^59,60^ hosted at Basel University Hospital.

#### Overall satisfaction

Overall satisfaction with therapy was assessed using a score between 0 = not at all satisfied and 10 = fully satisfied. Furthermore, women were asked if they would recommend the received care to other women with FGM/C.

#### Female Sexual Function Index

The FSFI^57^ is a self-report assessment of female sexual function in the previous 4 weeks. It consists of 19 items that assess 6 domains: desire, arousal, lubrication, orgasm, satisfaction, and pain. It has been shown to have good psychometric properties and clinical utility. In addition to the individual domains, a total score is calculated. It ranges from 2 to 36, with higher scores indicating more positive sexual function. In our study, the 4-week time was modified from 4 weeks to “since therapy.”

#### Genital pain

Genital pain was assessed using the FSFI and questions regarding pain symptoms during and outside of sexual intercourse before and after multidisciplinary care. We asked about the location of genital pain (e.g., generalized or localized: clitoris or other vulval regions), if genital pain was provoked or unprovoked, its time of onset, and the factors responsible for provoking such pain (e.g., touching or sex).

#### Female Genital Self-Image Scale

The FGSIS^55^ is a measurement instrument consisting of seven items for assessing female genital self-image. The questions relate to women’s feelings and beliefs about their own genitals. A total score is calculated using a 4-point response scale (strongly agree, agree, disagree, and strongly disagree). It provides a total score between 7 and 28, with higher scores indicating a more positive genital self-image. A positive genital self-image has been found to be associated with a positive body image.^61^

#### WHO-Five Well-Being Index

The WHO-5^56^ consists of five questions for assessing general mental well-being. For each question, scores from 0 to 5 can be given. By adding up the values for the answers, a total value between 0 and 25 is obtained. A low total value corresponds to low well-being.

#### Postoperative complications

Data on postoperative complications were collected through reviewing the participants’ medical records. No standard scale or tool for postoperative complications exists for CR and other surgeries following FGM/C. One possible approach to classify labial and clitoral reconstruction was proposed by Christopher et al. in a sample of 19 patients and distinguishes between “surgical site occurrences” (SSOs) (e.g., hematoma, seroma, impaired healing, and surgical site infection), “surgical site occurrences requiring procedural intervention,” and “long-term outcomes” (e.g., excessive scarring).^43^ Alternatively, a modified version of the widely used Clavien-Dindo classification according to the International Urogynecological Association (IUGA) and the American Urogynecological Society (AUGS) for cosmetic gynecologic surgery can be used.^62^ Each grade of the Clavien-Dindo classification^63^ is defined by the management of a specific postoperative complication. The complications of our study population were categorized according to both classifications. To facilitate the standardized reporting of postoperative complications after surgical treatment of FGM/C, further modifications were made to the Clavien-Dindo classification, which are described in Table 9.

### Statistical analysis

We present descriptive data on the patients’ sociodemographic characteristics, motivations to undergo therapy, therapy satisfaction, and outcomes: postoperative complications, sexual function, vulvar pain, and genital self-image. The primary outcome was overall satisfaction after the received care. To prevent missing data, all interviews were held in person or by telephone. Women with incomplete data to calculate a specific score were excluded from the analysis. The corresponding sample size is reported in each case. The data analysis was performed using R Statistical Software (v4.2.0; R Core Team 2022).^64^ In addition to the quantitative data collection, there were options for open answers to some questions. These were analyzed using thematic analysis based on keywords. Individual quotes from the study population are used in the publication to emphasize quantitative statements. No confirmatory tests were performed because validity could not be achieved due to the small sample size (*n* = 20). The study findings are reported according to the STROBE guidelines.^65^

### Patients’ clinical pathway

**Figure 2.**
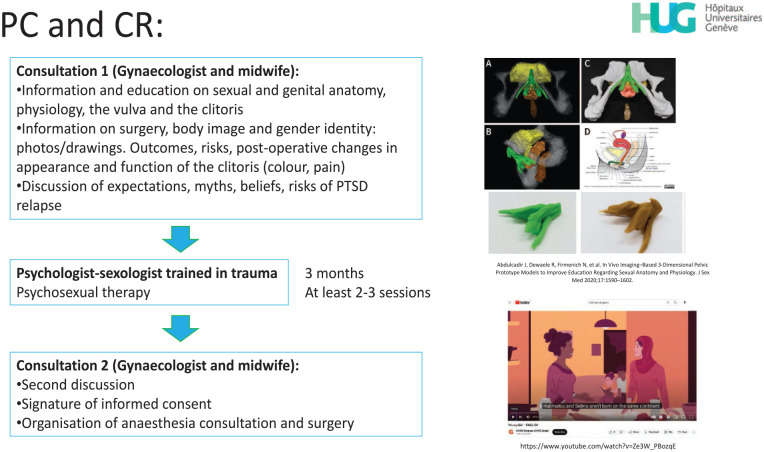
Summary of the consultations preceding possible CR. On the right, a 3D resource^70^ and video^74^ used with patients. CR: clitoral reconstruction.

**Figure 3.**
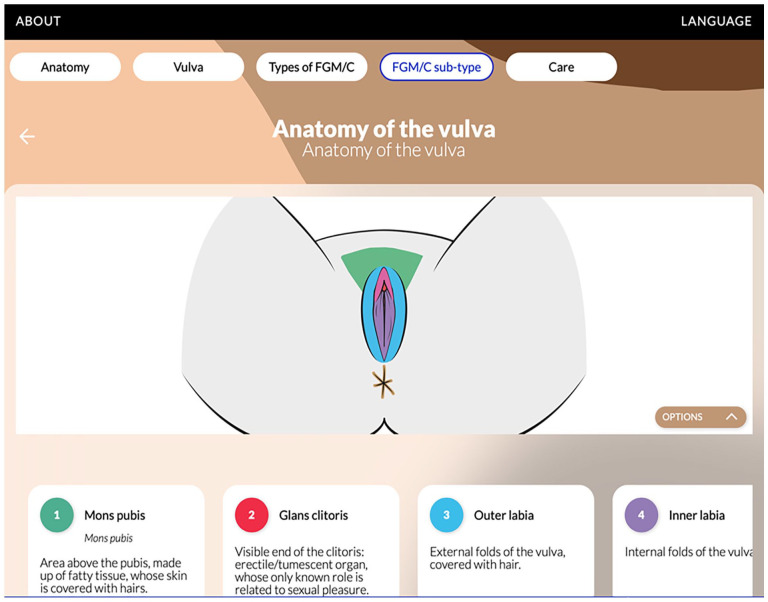
Web application offering information about female genital anatomy, FGM/C types, and care options.^69^ FGM/C: female genital mutilation/cutting.

Women requesting CR can be referred or can book themselves an appointment at the clinic under investigation. As described in Figure 2, they are first received by a gynecologist and surgeon certified in sexual medicine and trained in FGM/C and vulva disease (JA) together with a midwife (EM) certified in sexology. The gynecologist enquires about family, social, medical, sexual, and FGM/C history and discusses individual needs, questions, motivations for surgery, physical and psychological complaints, expectations from therapy, and other past traumatic events apart from FGM/C (e.g., rape, war, loss, migration trip, and forced or child marriage). The gynecologic exam is conducted with a colposcope, and the patient is invited to follow the exam and look at her genitals to understand her individual genital anatomy.^66^ According to patient needs, further information is provided using drawings, pictures, or videos showing vulvas with and without FGM/C,^67^ the anatomic diversity of genitals, and how the vulva looks before and after CR, immediately and in the long term.^67–69^ To do so, we use 3D pelvic and vulvar models,^70,71^ 3D magnetic resonance imaging-based clitoral models that represent genitals with or without FMG/C,^70,72,73^ a video summarizing the care,^74^ and a web application (see Figure 3) explaining vulvar anatomy, types of FGM/C, and the treatment options.^69^ The entire approach is culturally and trauma informed. This means that each step is explained and conducted in a safe space, the timing of each person is respected, their consent is renewed, and patients’ cultural values and beliefs are acknowledged.

#### Psychosexual care

After the first consultation, the patient is referred to a sex therapist and psychologist (NR) for psychological counseling and assessment with, when needed, sex and trauma therapy (e.g., eye movement desensitization and reprocessing). If necessary, a psychiatric sexologist is also available for supervision or to see the patient.

The duration and frequency of PC can vary depending on the individual’s needs, availabilities, and partner involvement. Usually, a general, psychological, and sexual history of the patient is taken by the sex therapist. Misconceptions that patients may have about sexuality or about her and others’ uncut anatomy (e.g., that women without FGM/C always experience spontaneous sexual desire and always have orgasms) are clarified. Information and advice about sexual health and specific support such as relaxation and home-based exercises to become familiar with the genitals are offered before surgery.

Patients are asked to discuss their expectations about CR with both the gynecologist and the psychologist, with a special emphasis on the occurrence and treatment of possible sexual complaints (e.g., regarding lubrication and sex toys^75^).

In general, PC takes 2–3 months before the patient is referred again to the surgeon for a second discussion, for signing the informed consent, and for planning the surgery. A report summarizing the PC is provided by the sex therapist to the surgeon, summarizing the care offered and confirming the absence of possible contraindications to surgery.

The psychosocial or psychiatric contraindications or conditions requiring preoperative resolution may be of a social nature—for example, an inability or unwillingness to stay away from work for at least 6 weeks post-operation, an unstable living or housing situation, or roommates, family members, or partners who do not know about the surgery—or of a psychological nature: for example, severe mental health conditions such as major depression, unrealistic expectations such as that marital problems will be solved with the surgery or dysmorphophobia. Physical or medical comorbidities such as immunodepression, smoking, or coagulopathy are assessed by the gynecologist surgeon and the anesthesiologist. Some contraindications are temporary and can be solved or managed by planning the surgery and the postoperative period at a better time.

#### Clitoral reconstruction: surgery

**Figure 4.**
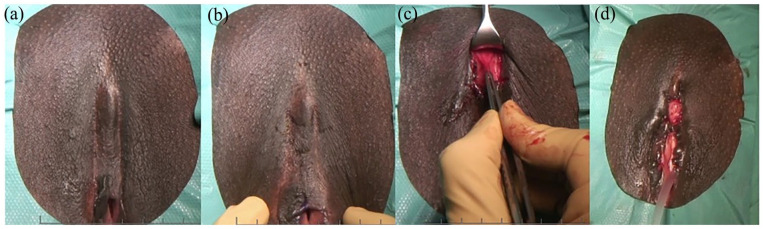
(a) FGM/C type IIIa with excision of the clitoris. (b) Omega dome flap drawn before incision according to O’Dey technique.^39,60^ (c) Clitoral reconstruction according to Foldès/Thabet technique: the superficial part of the suspensory ligament is visible. (d) Final appearance after clitoral reconstruction according to Foldès/Thabet technique combined with defibulation and reconstruction of the clitoral hood. FGM/C: female genital mutilation/cutting.

In our clinic, CR has been performed according to the Foldès/Thabet^40^ surgical technique (see Figure 4) since 2013. This involves the incision of the cutaneous scar tissue above the palpable body of the clitoris with the subsequent nonmicrosurgical dissection of the clitoral body while preserving the neurovascular bundle. The cutaneous incision used to be linear or lozenge shaped, as described by Foldès in 2004.^76^ In 2022, we started performing an omega-shaped incision that allows to reconstruct the clitoral prepuce as described by O’Dey.^33,77,78^ The suspensory ligament is partially sectioned to mobilize and pull the body of the clitoris, which will be stitched to the bulbocavernosus muscles. The sutures used are Monocryl 4.0. The surgery is conducted under general anesthesia with a laryngeal mask, under antibiotic prophylaxis (cefazoline 2 g or clindamycin 600 mg in case of allergies to cephalosporins), without an indwelling catheter, and usually as a day surgery. Patients who live far away or alone can spend one night at the hospital. The surgery lasts about 45–60 min. Since 2019, autologous platelet-rich plasma (A-PRP; BCT Regen-Lab^®^, Lausanne, Switzerland) can be injected intraoperatively into the clitoral body to promote faster healing and re-epithelialization. The injection of A-PRP seems useful in various indications to reduce postoperative pain and the chance of infection and to improve postoperative recovery.^35,36,79–81^ CR and the associated PC are fully refunded by the health insurance system in Switzerland, FGM/C being a code of the International Classification of Diseases since 2015.^82^ A-PRP BCT Regen-Lab is not refunded by health insurance, and costs 70 CHF for the patient if she wants to have it injected.

In our experience, postoperative pain starts to be more intense on day 7, not before. We do not perform local perioperative anesthesia or a pudendal block, and patients can easily go home on the same day as the surgery. They are seen on day 7, 30, 90, and 1 year. Patients go home with prescriptions to take 1 g of paracetamol four times a day, 400 mg of ibuprofen three times a day, and 30 mg of tramadol four times a day and to use paraffin gauze and lidocaine 2% gel. During the periodic post-op follow-up, according to the patient’s needs, we gradually decrease the painkillers, which are completely stopped between 2 and 3 months after the operation. In 11 years, no patient has needed additional pain treatment.

## Results

### Study population

We identified 34 medical files of French- or English-speaking women who asked for CR during their first consultation at the Geneva University Hospital FGM/C clinic between January 1, 2013 and November 30, 2021. Eight could not be reached and six declined to participate in the study, mainly due to a lack of time (Figure 1). Twenty women consented to be enrolled: 19 interviews were conducted in French and one in English. At the time of their first FGM/C consultation, the women were 38.55 ± 8.12 years old on average (range 19–50). They were all premenopausal.

As summarized in Table 3, most of the women had FGM/C type III (*n* = 10, 50%) involving the cutting of the clitoris. All the women stated that they had undergone FGM/C during childhood or adolescence (at a mean age of 7 ± 3 years old, range 1–13 years old). All were originally from sub-Saharan Africa. Only one of them was born in Europe, not in Switzerland. At the time of data collection, 60% (*n* = 12) had finished secondary school or had a university degree, 65% (*n* = 13) were single or divorced, 35% (*n* = 7) were married or in a relationship, 65% (*n* = 13) were employed or self-employed, and 90% (*n* = 18) had at least one child. All the women had spent at least 10 years in Switzerland.

**Table 3.** Sociodemographic characteristics of the total sample.

Origin	*n* = 20, *n* (%)
Africa	19 (95)
Ivory Coast	4 (20)
Somalia	4 (20)
Mali	3 (15)
Senegal	2 (10)
Guinea	2 (10)
Guinea-Bissau	1 (5)
Sierra Leone	1 (5)
Burkina Faso	1 (5)
Sudan	1 (5)
Europe (France)	1 (5)
Type of FGM/C	
Type I	1 (5)
Type II	9 (45)
Type III	10 (50)
Age at cutting (years)	
<5	2 (10)
5–10	14 (70)
>10	2 (10)
NA	2 (10)
Time spent in Switzerland (years)	
<10	0 (0)
10–15	9 (45)
16–21	4 (20)
22–27	5 (25)
>28	2 (10)
Education	
Early child education	4 (20)
Primary school	4 (20)
Secondary school	7 (35)
University degree	5 (25)
Profession	
Employed	13 (65)
Housewife	3 (15)
Internship	1 (5)
Unemployed	3 (15)
Children	
No children	2 (10)
1 child	7 (35)
2 children	5 (25)
>2 children	5 (25)
Relationship status	
Divorced	7 (35)
Single	6 (30)
Married	5 (25)
Relationship	2 (10)

FGM/C: female genital mutilation/cutting.

The women’s self-reported reasons behind their FGM/C included *conformity to social and cultural norms* (*n* = 18, 90%), *avoidance of hypersexuality* (*n* = 6, 30%), *purity* (*n* = 3, 15%), *marriageability* (*n* = 3, 15%), and *religion* (*n* = 1, 5%). None of them had been subjected to nonconsensual genital surgery for variations in their sexual development. Of the 20 women, 60% (*n* = 12) declared feeling uncomfortable talking about FGM/C with health professionals, partner(s), family members, or friends.

The women heard about CR from different sources: television (*n* = 7, 35%), their doctor or gynecologist (*n* = 7, 35%), friends (*n* = 6, 30%), the internet (*n* = 6, 30%), social networks (*n* = 5, 25%), their partner (*n* = 3, 15%), or a scientific study (*n* = 1, 5%). One-third of the women (*n* = 7, 35%) stated that they had requested an appointment immediately after hearing about the surgery, one-third (*n* = 7, 35%) had thought about it for several months or years before booking an appointment, and another third (*n* = 6, 30%) had waited for at least a decade before deciding to seek consultation. None of them requested labial reconstruction together with CR.

### Treatment received

Of the 20 women enrolled, 60% (*n* = 12) underwent CR in combination with PC, and 40% (*n* = 8) PC only. Among the 12 women who underwent CR in combination with PC, 6 (50%) received one A-PRP BCT Regen-Lab intraclitoral injection during surgery and 42% (*n* = 5) underwent defibulation at the same time.

Table 4 summarizes the reported motivations for seeking CR. We identified three main drivers of motivation in our study population: (1) to improve sexual response; (2) to be “repaired,” “normal,” or “empowered”; and (3) to treat vulvar pain. The most frequent motivations were to be “repaired” and to improve sexual response.

**Table 4.** Motivations for undergoing CR.

Motivation	Main motivation (single selection), *n* = 20, *n* (%)^a^	Other motivations (multiple selections possible), *n* = 20, *n* (%)
Improve sexuality	5 (25)	15 (75)
Be repaired^b^	6 (30)	13 (65)
Reduce vulvar pain	2 (10)	6 (30)

CR: clitoral reconstruction.

a Thirty-five percent (*n* = 7) of respondents did not provide a main motivation but gave equal weight to the other options specified.

b To feel repaired, normal, and empowered.

Of the 12 women who underwent CR, 75% (*n* = 9) indicated a main motivation: 42% (*n* = 5) wanted to be repaired, 17% (*n* = 2) to improve their sexuality, and 17% (*n* = 2) to relieve genital pain.

### Overall satisfaction with treatment received

The overall satisfaction with the care received was 8.95/10 ± 1.39 on average. The mean score was 8.57/10 ± 1.62 for the patients who received PC only and 9.17 ± 1.27 for those who received PC and CR.

**Figure 5.**
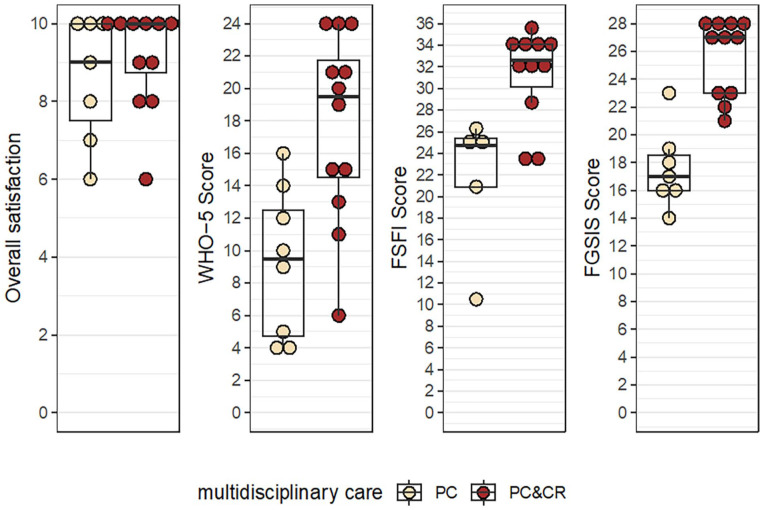
Therapy outcome of multidisciplinary care consisting either of PC or of PC&CR as measured by satisfaction, well-being, sexual response, and genital self-image. CR: clitoral reconstruction; PC: psychosexual care.

As shown in Table 5 and Figure 5, the women who underwent CR in combination with PC showed higher scores in overall satisfaction, the WHO-5, the FSFI, and the FGSIS than the women who underwent PC only.

**Table 5.** Therapy outcome by care received.

Outcome	Psychosexual care, mean (SD)	Psychosexual care and clitoral reconstruction, mean (SD)
Overall satisfaction	8.57 (1.62)	9.17 (1.27)
WHO-Five Well-Being Index	9.25 (4.62)	17.8 (5.77)
Female Sexual Function Index	21.56 (6.52)	31.22 (4.24)
Desire	2.85 (1.27)	4.70 (0.99)
Arousal	3.80 (0.98)	4.95 (0.63)
Lubrification	3.47 (1.92)	5.29 (1.04)
Orgasm	2.74 (1.69)	5.43 (0.84)
Satisfaction	4.07 (1.35)	5.67 (0.45)
Pain	4.17 (2.00)	5.37 (0.93)
Female Genital Self-Image Scale	17.60 (2.88)	25.60 (2.77)

WHO: World Health Organization; SD: standard deviation.

Of the 20 women, 95% (*n* = 19) stated that they would recommend undergoing multidisciplinary care to other women with FGM/C and thought that considering the possibility of surgical CR is essential; 85% (*n* = 17) thought that PC is an essential component of care.

### Reasons for not undergoing CR

The 8 women who decided not to receive surgery all stated that they were satisfied with PC. However, 62.5% (*n* = 5) of them declared that they might reconsider having the surgery one day in the future.

The reasons for not undergoing surgery were diverse. Two women stated it was not the right moment without further details; one was fully satisfied with PC; one abandoned the project of CR as she had a partner who feared her postoperative infidelity and hypersexuality; one could not be absent from work for more than 1 month after the operation; and one feared the risk of a possible negative outcome, particularly decreased clitoral sensitivity and chronic pain. Two women were in therapy for depression and were advised by the psychologist to undergo CR once their mental health was better, and then spontaneously abandoned the project of CR.

### Sexual function

At the time of the data collection, 95% (*n* = 19) of the women had been sexually active after the treatment. Among them, 53% (*n* = 10) had only had partnered sex, while 47% (*n* = 9) had practiced both masturbation and partnered sex. The person who had not been sexually active reported hypoactive desire before and after PC. Furthermore, she reported not having a partner at the time of data collection.

As shown in Figure 5 and Table 5, the FSFI (*n* = 16, 4 incomplete) shows a mean total score of 28.20 ± 6.69 after multidisciplinary care. The women who underwent CR in addition to PC had higher average total scores: 31.22 ± 4.24 versus 21.56 ± 6.52.

Of the total sample, 75% (*n* = 15) subjectively reported having sexual “problems” prior to multidisciplinary care, defined as hypoactive sexual desire or a lack of arousal, pleasure, or orgasm. After care, sexual “problems” were referred to by 35% (*n* = 7) of the women. Of those seven women, 85% (*n* = 6) did not undergo CR. One patient reported decreased sexual desire and pleasure after CR. However, this patient underwent several surgeries different from CR and suffered from deep dyspareunia before clitoral surgery due to uterine myomas. Four months before CR, she had undergone a total abdominal hysterectomy but kept experiencing deep dyspareunia when she resumed penetrative sexual intercourse. In addition, the laparotomic hysterectomy was complicated by an abdominal eventration treated with a second abdominal surgery 1 year after the hysterectomy with a self-reported major functional and aesthetic impact. She declined further medical advice or a referral at the time of the study.

The women with higher WHO-5 scores also showed higher FSFI and FGSIS scores, as shown in Table 6. Overall, 25% (*n* = 5) of the women had a low WHO-5 score (below 10). Among them, one woman had undergone PC and CR. Of the 75% (*n* = 15) of participants with a WHO-5 score above or equal to 10, 73% (*n* = 11) had undergone PC and CR.

**Table 6.** Well-being, genital self-image, sexual function, and arousal.

WHO-5 score	FGSIS, mean (SD)	FSFI, mean (SD)	FSFI arousal, mean (SD)
Women with WHO-5 score ⩾10 (*n* = 15)	23.40 (4.85)	28.97 (6.77)	4.67 (0.94)
Women with WHO-5 score <10 (*n* = 5)	18 (0.82)	22.80 (1.90)	3.8 (0.37)

FSFI: Female Sexual Function Index; FGSIS: Female Genital Self-Image Scale; WHO-5: WHO-Five Well-Being Index; SD: standard deviation.

### Vulvar pain

45% (*n* = 9) reported suffering from vulvar pain before care: one had spontaneous and provoked clitoral and vulvar pain, and eight provoked vulvar pain. Four out of these nine women underwent PC and CR (three with defibulation at the same time) with complete resolution of the pain. Among the remaining five women, one had defibulation and PC, and four had PC only. Three of the four who had PC only still reported suffering from deep and superficial dyspareunia at the time of the interview. Table 7 shows the characteristics of the pain before and after treatment.

**Table 7.** Vulvar pain in patients before and after treatment.

ID	Before treatment			Treatment	After treatment		
	Vulvar pain	Clitoral pain	Dyspareunia		Vulvar/clitoral pain	Dyspareunia	FSFI pain score
1	Provoked	—	Superficial	PC and CR and defibulation	—	—	5.2
2	Provoked	—	Deep	PC and CR	—	—	4.8
3	Unprovoked and provoked	Yes	Superficial	PC and CR and defibulation	—	—	5.3
4	Provoked	—	Superficial and deep	PC	—	Superficial and deep dyspareunia	1.6
5	Provoked	—	Superficial and deep	PC	—	Superficial and deep dyspareunia	3.2
6	Provoked	—	Superficial and deep	PC	—	Superficial and deep dyspareunia	NA
7	Provoked	—	Deep	PC	—	—	1.6
8	Provoked	—	Superficial	PC and CR and defibulation	—	—	3.6
9	Provoked	—	Superficial and deep	PC and defibulation	—	—	5.2

CR: clitoral reconstruction; PC: psychosexual care; FSFI: Female Sexual Function Index.

### Genital self-image

The mean FGSIS score at the time of data collection was 22.50 ± 4.88 out of a total of 28 points. As shown in Table 8, the women who underwent CR and PC registered a higher FGSIS mean total score than the women who did not undergo surgery: 25.60 ± 2.77 versus 17.60 ± 2.77.

**Table 8.** Women with positive or negative genital self-image before and after undergoing PC only or PC in combination with CR.

PC (*n* tot = 8)	Yes, *n* (%)	No, *n* (%)
Before care I had a positive genital self-image	1 (12.5)	7 (87.5)
After care I had a positive genital self-image	4 (50)	4 (50)
PC and CR (*n* tot = 12)		
Before care I had a positive genital self-image	4 (33.33)	8 (66.67)
After care I had a positive genital self-image	11 (91.67)	1 (8.33)

CR: clitoral reconstruction; PC: psychosexual care.

Of the 20 women, 25% (*n* = 5) reported having a positive genital self-image prior to therapy. PC alone increased the percentage of women with a positive genital self-image from 12.5% to 50% (*n* total = 8), and PC in combination with CR increased it from 33.33% to 91.67% (*n* total = 12) (see Table 8).

### Postoperative complications and subjective adverse outcomes of CR

According to Christopher et al.,^43^ the immediate complication rate in our study was 16.67% (*n* = 2) with two SSOs. In addition, there were four further complications or subjective adverse outcomes that could not be classified according to Christopher et al. According to the Clavien-Dindo classification modified by IUGA, AUGS, and the authors, the above-mentioned events (one grade 0, two grade I, one grade II, two not listed) occurred among five patients. An overview of the complications and subjective adverse outcomes is shown in Table 9, and the classification is given in parentheses in the text. One patient presented nonsevere postoperative bleeding within 2 h of the surgery (grade I, SSO) and was treated with a compressive dressing and 1 g of intravenous tranexamic acid, with no further surgical intervention, no hematoma, and no longer hospital stay. One woman presented with a wound infection on day 6 (grade I, SSO), which was treated with a 5-day course of oral metronidazole. However, she only took paracetamol as a pain killer, and the postoperative pain in the genital area triggered memories of the traumatic experience of genital mutilation with a relapse of symptoms of posttraumatic stress disorder. She was successfully treated by cognitive behavioral therapy and pharmacological therapy (grade II “pt”) with a positive long-term outcome in terms of satisfaction and psychosexual health. This case was published by Abdulcadir et al.^83^

**Table 9.** Complications according to the classification of cosmetic gynecology; revisions and modifications for surgical treatment of FGM/C (modifications by the authors underlined).

Grade	Definition	Number of events
Grade 0 (revision rate)	Surgical revision for aesthetic indication	0
Suffix “a”	Asymmetry, contour irregularities	0
Suffix “o”	Overcorrection	
Suffix “u”	Undercorrection	1
Suffix “s”	Scarring	
Grade I	Transient or minor complications not requiring significant medial intervention	
Suffix “abx”	Antibiotics	1
Suffix “inj”	Injection Examples: i.v. tranexamic acid, steroid injection for pain, edema, seroma, superficial wound separation, granulation tissue	1
Grade II	Moderate complications requiring either prolonged or substantial medical intervention, including hospitalization	
Suffix “pt”	Physical therapy and/or psychosexual therapy	1
Suffix “b”	Blood transfusion Examples: dyspareunia requiring pelvic floor physical therapy, blood transfusion for anemia, hospitalization for postoperative infection, deep vein thrombosis	
Grade III	Complication requiring surgical intervention Examples: hematoma, hemorrhage, necrosis requiring resection	
Grade IV	Life-threatening complication	
Suffix “d”	Death of patient Examples: sepsis, myocardial infraction, organ failure	0
Grade NL	Complication profile not listed or clearly reported	2

FGM/C: female genital mutilation/cutting.

One patient reported decreased sexual desire and pleasure since CR (grade not listed). Two women reported not being fully satisfied with the appearance of their clitoris due to a clitoral bulge judged to be insufficiently visible. One of these two requested a second clitoral surgery, which was performed more than 1 year after follow-up with counseling and PC (grade 0). After the second operation, she was fully satisfied with both her appearance and sexual function. The other woman did not want a second surgery and reported that even though the bulge was not prominent, her sexual functioning and sensitivity had improved after CR and PC (grade not listed).

## Discussion

Our data show that PC in combination with CR led to high satisfaction rates in the study population: 8.95/10 ± 1.39. The high satisfaction is evident in a high recommendation rate of 95% (*n* = 19), as one of the women expressed:

“Le jour de l’opération est comme un deuxième anniversaire pour moi.” (The day of the surgery is like a second birthday for me.)

The women who underwent CR in combination with PC showed higher mean scores for overall satisfaction, general psychological well-being (WHO-5), sexual function (FSFI), and genital self-image (FGSIS) than the women who only underwent PC, as described in Figure 5. Nevertheless, the overall satisfaction of the women who received PC only was very good and diverged less than the other parameters assessed.

**Figure 6.**
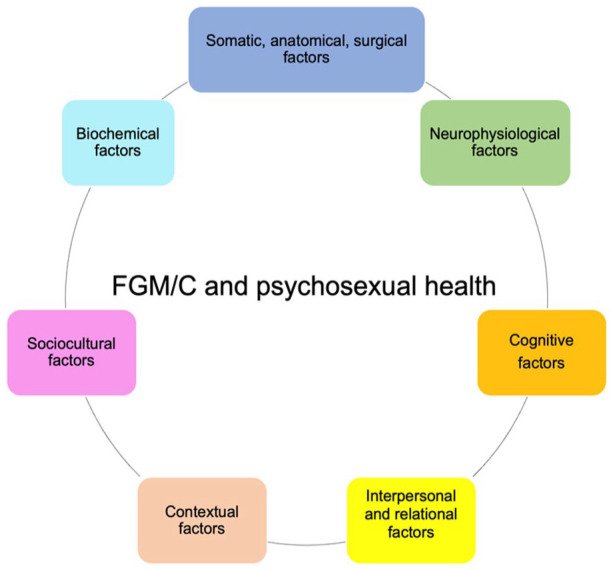
The complex interplay between FGM/C and psychosexual health. FGM/C: female genital mutilation/cutting.

Prior literature on CR has shown positive effects on sexual function.^30,34,39–41,47,51^ While previous authors have found that CR combined with PC can improve sexual distress, psychopathology, and sexual function,^84^ our study identified a subjective improvement after both PC alone and PC combined with CR. As illustrated in Figure 6, PC is crucial for women undergoing CR, as biopsychosocial factors need to be addressed to promote psychosexual health and psychosocial well-being. Psychological well-being impacts sexual response, particularly arousal^85–87^; indeed, in our sample, women with lower psychological well-being also had lower FSFI and FGSIS scores (see Table 6). Recently, a history of physical abuse was found to be associated with chronic clitoral pain after CR.^88^ And clitoral knowledge and less gendered sexual scripts, both potentially improved by PC, have been found to be associated with higher pleasure and orgasm experience.^66^

In contrast to the data of Paslakis et al.,^53^ which showed an improvement in sexual distress but no improvement in sexual function following surgery, our data showed a mean total FSFI score of 21.56 ± 6.52 after PC only and of 31.22 ± 4.24 after PC and CR. This is similar to the median score of 29 (interquartile range: 24–34) described by Vital et al. after CR.^54^ Based on the FSFI cutoff value of 26.55,^89^ 43% (*n* = 7, four incomplete) of the women in our sample were found to have sexual dysfunction after care, which approximates the general population prevalence of 40% to 50%.^90^ Of the seven women with sexual dysfunction according to the FSFI, six underwent PC alone and one PC combined with CR. We cannot conclusively determine whether these results are attributed to the effect of combining CR with PC, which improves sexual function and psychological well-being more than PC alone, or if individuals with sexual dysfunction and low psychological well-being are less inclined to pursue surgery. Future studies should try to assess both subjective and objective outcomes such as changes in clitoral sensitivity and pain threshold. A recent study showed that the Foldès CR technique can significantly improve clitoral sensitivity based on an assessment with Semmes-Weinstein monofilaments.^91^ Whether such increased sensitivity correlates with more sexual pleasure deserves to be studied further.

Subjective improvement in genital self-image occurred in our sample both with PC alone and with PC in combination with CR (Table 8). Consistent with the findings of Berg et al., who observed postoperative clitoral visibility in 75% of cases,^48^ 83.3% of our sample was satisfied with their own clitoral bulging. Nonetheless, patients’ subjective genital self-image and surgeons’ perceptions of anatomical aesthetics and norms diverge. In their study, Abramowicz et al. noted that while the postoperative results were deemed satisfactory in all 26 cases by the surgeon, only 58% of the patients judged the postoperative clitoral appearance as “normal” according to their own criteria. Regardless, 96% reported enhanced subjective genital anatomy.^49^

Both those who underwent PC alone and those who received CR in combination with PC reported an improvement in pain symptoms (see Table 7), although the subjectively perceived efficacy of the methods varied widely. Moreover, the accurate quantification of pain was difficult to measure due to partial scoring by the FSFI and other pain assessment questions, leading to inconsistencies in the results (see, e.g., ID 7 in Table 7). To reduce FGM/C-related vulvar pain, several treatment options exist, including physiotherapy, vulvar scar correction, defibulation, CR, and vulvar reconstruction.^92^ Since these therapeutic interventions are often employed in combination, it is difficult to identify the impact of any single intervention on pain. In our population, defibulation, which is known to reduce dyspareunia,^93^ was performed simultaneously on five women. Vulvar pain and dyspareunia can be caused and influenced by diverse neurobiological, muscular, and psychosexual factors other than FGM/C,^94^ and they must also be addressed in women and girls with FGM/C (see Figure 6).^95,96^ Some authors have even mentioned a possible altered pain perception due to neurobiological changes,^95^ resulting in more complex pain assessment after FGM/C.

The Foldès/Thabet technique in CR seems reproducible. When associated with PC and performed in specialized or dedicated settings, it has shown favorable outcomes and minor complications.^48,97^ Our observed complication rate of 16.67% (*n* = 2) aligns with the 15.4% reported by Berg et al.’s meta-analysis^48^ but is higher than the 3% reported in the scoping review by Almadori et al.^51^ To facilitate the standardized reporting of postoperative complications following the surgical treatment of FGM/C, we have adapted the Clavien-Dindo classification of cosmetic gynecology for potential use (see Table 9). Based on this classification, not being subjectively satisfied with aesthetic appearance and/or function will also be categorized as subjective adverse outcomes. In our sample, two patients were not fully satisfied after treatment with the amount of bulging of the clitoris but were satisfied with functioning. It would be useful to have a standardized and reproducible definition of satisfaction for assessing based on pre-op expectations and post-op satisfaction.

Little is known about the outcome of the surgery in (peri-)menopausal women and the possible recommendations of hormonal therapy, which could be beneficial before and after surgery. In our experience, women increasingly become interested in CR after the age of 45, once their children have grown up, maybe after a divorce or a separation, when their job is secure, and, to quote many of our patients, “there is time to think more about themselves.”

In previous studies, up to 36% of women who initially requested CR finally underwent surgical therapy after multidisciplinary care.^18^ In our study, 60% underwent CR. This discrepancy might be explained by our study design, as those who were initially not seeking surgery and received PC alone were not included. All our participants came to the clinic for CR. In addition, our clinic is the only Swiss FGM/C clinic in a public hospital that offers PC and CR at no cost, and the women who come or are referred for CR are highly motivated for surgery and sometimes already selected by the providers who refer them. Opting for PC alone was influenced by factors that partially coincide with those described in the literature: unrealistic surgical expectations, satisfaction from PC, procedural and postoperative pain apprehensions, and balancing considerations.^18,19^

Specific subjective symptoms, together with gender identity, societal norms, stigmatization, and attitudes toward FGM/C^2,98–102^ strongly influence decisions regarding CR.^32^ Our participants’ narratives demonstrate how partner dynamics, societal norms, and personal history converge to influence decisions for or against therapy. As already mentioned in our previous papers, we believe that biopsychosocial factors should be addressed with trauma-sensitive and culturally informed care of patients who are being consulted for CR.^103^ Such factors impact not only their sexual function but also their treatment decisions and the outcomes of such treatments. The concept of symbolic reconstruction as the “rectification of stigma, injustice, and past negative sexual and social experiences” resonated with our participants’ narratives.^31^ These feelings were exemplified by statements like:

“Il [ex-partner] est parti parce que je suis excisée.” (He [ex-partner] left because I was cut).

“Je veux être comme tout le monde : esthétique et fonctionnelle.” (I want to be like everyone else: aesthetic and functional).

A systematic review investigating the impact of FGM/C on sexual functioning, which primarily included studies conducted in Western countries, showed a higher prevalence of dyspareunia, diminished sexual desire, and lower sexual satisfaction among women with FGM/C.^11,104^ On the other hand, Ahmadu describes the possible onset of sexual dysfunction post-migration,^12^ which might be explained by the influence of societal discourses about physiological sex and individual gender identity.^105^ The stigmatization and taboos around FGM/C were evident in our patients’ discomfort about discussing FGM/C with friends, family, and partners. Some women specifically stated discomfort with FGM/C post-migration:

“Je me suis sentie normal avant venir en Suisse. Ç’a commencé de piquer depuis que je suis en Suisse.” (Before I came to Switzerland, I felt normal. Since I’ve been in Switzerland, it’s started to sting.)

“Après passer les frontières et venir en Suisse je me suis sentie mal.” (After crossing the borders and coming to Switzerland, I felt bad.)

### Strengths and limitations

The limitations of our study include the limited sample size, the absence of a power analysis, selection bias at the time of the interview, and the fact that the postoperative interview was performed at different time points after intervention (3 months to 5 years), which could have led to different types of response biases, such as acquiescence bias or choice-supportive bias, meaning that the chosen procedure was perceived as better than it actually is. To strengthen the generalizability of the results, a prospective multicenter study would be ideal.

The strengths of this study include the comprehensive and precise biopsychosocial assessment of the participants through tools that can be replicated in further studies in different settings. Furthermore, the interview was not conducted by the provider who performed the surgery, the clinical care, and the follow-up.

## Conclusion

Our study shows positive outcomes of trauma-sensitive and culturally informed PC and CR using the Foldès/Thabet technique with improved sexual function, psychological well-being, and genital self-image and few minor complications. Tailoring the care to individual motivations and decision-making processes can yield high satisfaction, whether surgery is involved or not. This study can also facilitate reproducing our care pathway in other clinics since it describes in detail the multidisciplinary care of women with FGM/C. The data collection instruments used can be reused or adapted for future research.

## Supplemental Material

sj-docx-1-whe-10.1177_17455057251315814 – Supplemental material for Clitoral reconstruction and psychosexual care after female genital mutilation/cutting: Assessment of multidisciplinary care Supplemental material, sj-docx-1-whe-10.1177_17455057251315814 for Clitoral reconstruction and psychosexual care after female genital mutilation/cutting: Assessment of multidisciplinary care by Muriel Meyer, Gideon Sartorius and Jasmine Abdulcadir in Women's Health

sj-docx-2-whe-10.1177_17455057251315814 – Supplemental material for Clitoral reconstruction and psychosexual care after female genital mutilation/cutting: Assessment of multidisciplinary care Supplemental material, sj-docx-2-whe-10.1177_17455057251315814 for Clitoral reconstruction and psychosexual care after female genital mutilation/cutting: Assessment of multidisciplinary care by Muriel Meyer, Gideon Sartorius and Jasmine Abdulcadir in Women's Health

sj-docx-3-whe-10.1177_17455057251315814 – Supplemental material for Clitoral reconstruction and psychosexual care after female genital mutilation/cutting: Assessment of multidisciplinary care Supplemental material, sj-docx-3-whe-10.1177_17455057251315814 for Clitoral reconstruction and psychosexual care after female genital mutilation/cutting: Assessment of multidisciplinary care by Muriel Meyer, Gideon Sartorius and Jasmine Abdulcadir in Women's Health

sj-docx-4-whe-10.1177_17455057251315814 – Supplemental material for Clitoral reconstruction and psychosexual care after female genital mutilation/cutting: Assessment of multidisciplinary care Supplemental material, sj-docx-4-whe-10.1177_17455057251315814 for Clitoral reconstruction and psychosexual care after female genital mutilation/cutting: Assessment of multidisciplinary care by Muriel Meyer, Gideon Sartorius and Jasmine Abdulcadir in Women's Health

## Declarations

**Ethics approval and consent to participate:** The study was conducted in accordance with the ethical principles of the Declaration of Helsinki and was approved by the institutional review board (Geneva Commission cantonale d’éthique de la recherche, protocol number 2021-01190). All participants signed an informed consent after receiving detailed information and clarifications of any questions concerning the study.

**Consent for publication:** The informed consent document that was signed by all the participants included consent for publication.

**Availability of data and materials:** Not applicable for data; questionnaire in the Supplemental Appendix.
