# Supplementary material for: Clitoral reconstruction and psychosexual care after female genital mutilation/cutting: Assessment of multidisciplinary care
Source: Womens Health (Lond). 2025 Mar 31;21:17455057251315814. doi: 10.1177/17455057251315814 (PMC11960189; doi:10.1177/17455057251315814)
Supplement: sj-docx-1-whe-10.1177_17455057251315814 – Supplemental material for Clitoral reconstruction and psychosexual care after female genital mutilation/cutting: Assessment of multidisciplinary care [file sj-docx-1-whe-10.1177_17455057251315814.docx]

**Questionnaire**

Il s’agit d’un questionnaire de questions à choix multiples concernant l’étude : **La reconstruction clitoridienne (RC) chez les patientes atteintes de MGF: Évaluation de la prise en charge multidisciplinaire**

L’interview est structurée en 1h environ.

**Vos données socio-démographiques**

1. **Quel âge avez-vous ? … ans (une seule réponse)**
   - 18-24 ans
   - 25-29 ans
   - 30-34 ans
   - 35-39 ans
   - 40-44 ans
   - 45-49 ans
   - >50 ans
2. **Quel est votre pays de naissance ? (une seule réponse)**
   - Somalie
   - Erythrée
   - Ethiopie
   - Soudan
   - Mali
   - Guinée
   - Autre :
3. **De quel pays est originaire :**

**Votre mère ? (une seule réponse)**

- - Somalie
  - Erythrée
  - Ethiopie
  - Soudan
  - Mali
  - Guinée
  - Autre :

**Votre père (une seule réponse)**

- - Somalie
  - Erythrée
  - Ethiopie
  - Soudan
  - Mali
  - Guinée
  - Autre :

1. **Quelle(s) langue(s) parlez-vous ? (Plusieurs réponses possibles)**
   - Français
   - Anglais
   - Tigrinya
   - Somalien
   - Amharique
   - Arabe
   - Autre :
   - Dans quelle langue êtes-vous la plus à l’aise ?
2. **Depuis combien de temps vivez-vous en Suisse ? … ans/moins (une seule réponse)**
   - <6 mois
   - 6 mois – 1 an
   - 1-2 ans
   - 2-5 ans
   - 5-10 ans
   - >10 ans
3. **Quel est votre état civil ? (une seule réponse)**
   - Célibataire
   - Mariée
   - Divorcée/séparée
   - Veuve
   - Concubinage
4. **Depuis combien de temps ? … ans/moins (une seule réponse)**
   - <6 mois
   - 6 mois – 1 an
   - 1-2 ans
   - 2-5 ans
   - 5-10 ans
   - >10 ans
5. **Avez-vous des enfants ? (une seule réponse par section)**
   - Oui
   - Non

**Filles ?**

- - 0
  - 1
  - 2
  - $\geq$3

**Garçons ?**

- - 0
  - 1
  - 2
  - $\geq$3

1. **Avez-vous des enfants nés en Suisse ? (une seule réponse)**
   - Oui
   - Non
2. **Quel est votre niveau plus haut d’études ? (une seule réponse)**
   - Je n’ai pas été scolarisée
   - École primaire
   - Études secondaires partielles
   - Diplômée d’études secondaires
   - Premières années d’Université
   - Diplômée d’Université ou degré supérieur
3. **Quel est votre situation professionnelle ? (Plusieurs réponses possibles)**
   - Etudiante
   - En cours de français
   - Employée
   - Femme au foyer
   - Sans emploi
   - Retraitée
   - Inapte/invalide
   - Autre :

**Concernant votre excision et les MGF en général**

1. **Est-ce que vous vous sentez en mesure d’en parler : (une seule réponse par section)**

**Avec le corps médical ?**

- - Oui
  - Non

**Avec votre partenaire ?**

- - Oui
  - Non

**Avec vos proches ?**

- - Oui
  - Non

1. **Quel âge aviez-vous quand votre excision a été pratiquée ? (une seule réponse)**
   - Naissance ou quelque jours/mois après
   - <5 ans
   - 5-10 ans
   - 10-15 ans
   - 15-20 ans
   - >20 ans
   - Je ne sais pas/plus
2. **Pour quelles raisons on a décidé de pratiquer votre excision selon vous ? (Plusieurs réponses possibles)**
   - Pour devenir femme
   - Pour devenir pure
   - Pour être respectée
   - Pour pouvoir se marier un jour
   - Pour être dans la norme sociale et culturelle de votre communauté
   - Pour être plus belle
   - Pour empêcher l’hypersexualité
   - Pour être plus propre
   - Pour ne pas subir de jugement
   - Pour être protégée
   - Autre
   - Je ne sais pas / plus
3. **Comment décrieriez-vous votre expérience au moment de l’excision ? (Plusieurs réponses possibles)**
   - Positive
   - Négative
   - Neutre
   - Belle
   - Moche
   - Indolore
   - Douloureuse
   - Autre :
   - Je ne m’en rappelle pas
4. **Je me suis sentie : (Plusieurs réponses possibles)**
   - Courageuse
   - Fière
   - Honorée
   - Célébrée
   - Trahie
   - Seule
   - Fâchée
   - Heureuse
   - Malheureuse
   - Normale
   - Anormale
   - Confiante
   - Adulte
   - Apeurée
   - Autre :
   - Je ne m’en rappelle pas
5. **Qu’est-ce que vous ressentez à présent, d’avoir été excisée ? (Plusieurs réponses possibles)**
   - Respectable
   - Fière
   - Plus propre
   - Libre
   - En colère
   - Honteuse
   - Dégoutée
   - Coupable
   - Normale
   - Différente
   - Anormale
   - Autre :

**Concernant votre santé avant votre RDV à la consultation « MGF » :**

1. **Avant les soins quels symptômes aviez-vous ? (Plusieurs réponses possibles)**

- Pas de symptômes
- Urinaires (infections, obstruction du flux …)
- Douleur génitale en dehors des rapports sexuels
- Douleur génitale pendant les rapports sexuels
- Psychologiques (peur, honte, culpabilité, traumatisme, …)
- Génitaux (kystes, démangeaisons récurrentes, …)
- Sexuels (manque de désir ou plaisir, …)
- Pas de sexualité
- Autres :

1. **Étaient-ils liés selon vous à l’excision ? (une seule réponse)**
   1. Oui
   2. Non
2. **Avez-vous déjà rencontré des difficultés sexuelles ? (Plusieurs réponses possibles)**

- Non
- Oui. Si oui les quelles :
- Saignements durant les rapports
- Incapacité psychologique à avoir des rapports sexuels
- Incapacité physique à avoir des rapports sexuels
- Manque de plaisir durant l’acte sexuel
- Rapports sexuels retardés / repoussés
- Douleur durant les rapports
- Manque de désir
- Blessures sur les zones génitales durant les rapports
- Manque de lubrification
- Incapacité d’avoir un orgasme
- Autre :

1. **Avant les soins multidisciplinaires j’avais un sentiment positif concernant mes organes génitaux (une seule réponse)**

- 4= tout à fait d'accord
- 3= d'accord
- 2= pas d'accord
- 1= Pas du tout d'accord

**Concernant votre satisfaction des soins à la consultation « MGF » aux HUG (prise en charge psychosexuelle et éventuellement reconstruction clitoridienne).**

1. **En quoi ont consisté les soins multidisciplinaires dans votre cas ? (une seule réponse)**

- Suivi psychosexuel ?
  - 1. Combien de consultations avec un/une gynécologue?
    2. Combien de consultations avec un/une psychologue sexologue ?
- Suivi psychosexuels et reconstruction clitoridienne

1. **Qu'attendiez-vous/attendez-vous/espériez-vous du traitement chirurgical? * (texte libre)**
2. **Vos attentes sont-elles été satisfaites par la thérapie chirurgicale? * (une seule réponse)**

- Oui, totalement
- Oui, partiellement
- Non, pas du tout
- Pourquoi ? (texte libre)

1. **Vos attentes sont-elles été satisfaites par la thérapie psychosexuelle? (une seule réponse)**

- Oui, totalement
- Oui, partiellement
- Non, pas du tout
- Pourquoi ? (texte libre)

1. **Dans quelle mesure êtes-vous satisfaite de la thérapie multidisciplinaire sur une échelle de 0 à 10 (0=pas du tout satisfaite, 10=tout à fait satisfaite) ? (une seule réponse)**
2. **Pensez-vous que les soins psychosexuels ont permis une amélioration générale : (une seule réponse par section) (0=pas du tout d'accord, 10=tout à fait d'accord**

- De votre santé ? (0-10)
- De votre sexualité ? (0-10)
- De votre image génitale ? (0-10)
- Des douleurs génitales pendant les rapports sexuels? (0-10)
- Des douleurs génitales en dehors des rapports sexuels? (0-10)

1. **Pensez-vous que la reconstruction clitoridienne a permis une amélioration générale : * (une seule réponse par section) (0=pas du tout d'accord, 10=tout à fait d'accord**

- De votre santé ? (0-10)
- De votre sexualité ? (0-10)
- De votre image génitale ? (0-10)
- Des douleurs génitales pendant les rapports sexuels? (0-10)
- Des douleurs génitales en dehors des rapports sexuels? (0-10)

1. **Après la reconstruction clitoridienne quelle image avez-vous de vous-même ? (Plusieurs réponses possibles) ***

- Respectueuse
- Fière
- Propre
- Libre
- Sexy
- Normale
- Belle
- Complète
- En colère
- Honteuse
- Dégoutée
- Coupable
- Avec des regrets
- Je ne remarque pas de changement
- Autre :

1. **Aviez/avez-vous des craintes de la chirurgie ?* (une seule réponse)**

- Oui
- Non
- **Si oui : Lesquelles ? Étaient-elles fondées ? (texte libre)**

1. **Conseilleriez-vous votre suivi à d’autres femmes excisées ? (une seule réponse)**
   - Oui
   - Non
2. **Pensez-vous que les soins psychosexuels sont essentiels pour les femmes excisées ? (une seule réponse)**
   - Oui
   - Non
3. **Pensez-vous que la reconstruction clitoridienne est essentielle pour les femmes excisées ? (une seule réponse)**
   - Oui
   - Non
4. **Pourquoi avez-vous finalement décidé de ne pas faire la reconstruction clitoridienne ? (Plusieurs réponses possibles) ****

- Autres attentes en matière de reconstruction clitoridienne
- Besoins satisfaits par les soins psychosexuels
- Contre-indications médicales
- Circonstances sociales
- Levé de tabou
- Peur de l'opération
- Incertitude quant au résultat
- Sentiment de ne pas être comprise
- Nécessité d'une réflexion plus approfondie
- J’ai été opéré ailleurs
- Priorité ailleurs
- Parcours trop long
- Peur des douleurs
- Peur que ma sexualité change en pire
- Je n’ai pas renoncé, je suis juste en train d’attendre le bon moment
- Autres

1. **Qu'est-ce qui vous a le plus aidé personnellement ? (Plusieurs réponses possibles)**

- Parler
- Avoir une personne de contact
- Éducation sur la fonction et l'anatomie des organes génitaux féminins
- Support psychologique
- Traitement chirurgical
- Autres

1. **Y va-t-il des choses qui, selon vous, ont manqué dans ou pendant vos soins ? (texte libre)**
2. **Votre partenaire sait-il qu'il y avait des consultations aux HUG ? (une seule réponse)**

- Oui
- Non
- **Si oui, quelle est son attitude concernant les consultations ? (plusierus réponses possibles) :**
- Positive
- Negative
- Fier
- Méchant
- Triste
- Autre :

**Concernant votre motivation pour consulter aux HUG**

1. **Quelle était votre motivation pour la reconstruction clitoridienne ? (Plusieurs réponses possibles)**

- Douleur génitale pendant les rapports sexuels
- Douleur génitale en dehors des rapports sexuels
- Reconstruction de mon identité
- Obtenir une réparation
- Améliorer mon image corporelle/génitale
- Améliorer ma sexualité
- Se sentir comme une femme à part entière (normalité)
- Être comme les autres femmes (égalité)
- Récupérer ce qui m'a été pris (justice)
- Autre :

1. **Y avait-il une motivation principale pour vous ? (une seule réponse)**

- Oui
- Non

**Si oui, laquelle ? (texte libre)**

1. **Comment avez-vous découvert les soins multidisciplinaires / la reconstruction clitoridienne aux HUG ? (Plusieurs réponses possibles)**

- Par des amies
- Par un médecin (gynécologie, médecin de famille, …)
- Par mon partenaire
- Sur internet
- Sur les réseaux sociaux
- Autre :

1. **Combien de temps avez-vous pensé à consulter aux HUG avant de prendre rendez-vous ?**

**Concernant votre santé actuellement**

1. **Comment décririez-vous votre santé psychique ? (Plusieurs réponses possibles)**

- Bonne
- Mauvaise
- Trouble psychique connu, à savoir :

1. **Actuellement quels symptômes avez-vous ? (Plusieurs réponses possibles)**

- Urinaires (infections, obstruction du flux …)
- Douleur génitale pendant les rapports sexuels
- Douleur génitale en dehors des rapports sexuels
- Psychologiques (peur, honte, culpabilité, traumatisme, …)
- Génitaux (kystes, démangeaisons récurrentes, …)
- Sexuels (douleurs, manque de plaisir, …)
- Pas de sexualité
- Pas de symptômes
- Autres :

1. **Sont-ils liés selon vous (une seule réponse par section)**

**à l’excision ?**

- 1. Oui
  2. Non

**à la chirurgie ? ***

- 1. Oui
  2. Non

1. **Actuellement avez-vous des difficultés sexuelles ? (Plusieurs réponses possibles)**

- Non
- Oui. Si oui lesquelles :
- Saignements durant les rapports
- Incapacité psychologique à avoir des rapports sexuels
- Incapacité physique à avoir des rapports sexuels
- Manque de plaisir durant l’acte sexuel
- Rapports sexuels retardés / repoussés
- Douleur durant les rapports
- Manque de désir
- Blessures sur les zones génitales durant les rapports
- Manque de lubrification
- Incapacité d’avoir un orgasme
- Autre :

1. **Avez-vous un clitoris visible depuis la reconstruction clitoridienne ? ***

- Oui
- Non

**Well-being: Indice (en cinq points) de bien-être de l’OMS (1999)**

**Au cours des deux dernières semaines**

1. **Je me suis senti(e) bien et de bonne humeur**

- 5= Tout le temps
- 4= La plupart du temps
- 3= Plus de la moitié de temps
- 2= Moins de la moitié du temps
- 1= De temps en temps
- 0= Jamais

1. **Je me suis senti(e) calme et tranquille**

- 5= Tout le temps
- 4= La plupart du temps
- 3= Plus de la moitié de temps
- 2= Moins de la moitié du temps
- 1= De temps en temps
- 0= Jamais

1. **Je me suis senti(e) plein(e) d’énergie et vigoureux(se)**

- 5= Tout le temps
- 4= La plupart du temps
- 3= Plus de la moitié de temps
- 2= Moins de la moitié du temps
- 1= De temps en temps
- 0= Jamais

1. **Je me suis réveillé́(e) en me sentant frais(che) et dispos(e)**

- 5= Tout le temps
- 4= La plupart du temps
- 3= Plus de la moitié de temps
- 2= Moins de la moitié du temps
- 1= De temps en temps
- 0= Jamais

1. **Ma vie quotidienne a été remplie de choses intéressantes**

- 5= Tout le temps
- 4= La plupart du temps
- 3= Plus de la moitié de temps
- 2= Moins de la moitié du temps
- 1= De temps en temps
- 0= Jamais

**FSFI**

Instructions : Les questions suivantes portent sur vos sentiments et vos réactions sur le plan sexuel au cours des 4 dernières semaines. Veuillez répondre à ces questions aussi sincèrement et clairement que possible. Vos réponses resteront strictement confidentielles. Lorsque vous répondrez aux questions, tenez compte des définitions suivantes :

L'activité sexuelle peut comprendre les caresses, les préliminaires, la masturbation et la pénétration vaginale.

Le rapport sexuel se définit comme la pénétration (l'introduction) du pénis.

La stimulation sexuelle comprend, par exemple, les préliminaires avec un partenaire, la masturbation et les fantasmes sexuels.

Le désir sexuel est un sentiment qui comprend le désir d'avoir une activité sexuelle, le fait d'être réceptive aux avances sexuelles d'un partenaire et d'avoir des pensées ou des fantasmes à propos de l'acte sexuel.

1. **Au cours des 4 dernières semaines, avez-vous ressenti un désir sexuel ?**
   - 5=Presque toujours ou toujours
   - 4=La plupart du temps (plus d'une fois sur deux)
   - 3=Parfois (environ une fois sur deux)
   - 2=Rarement (moins d'une fois sur deux)
   - 1=Presque jamais ou jamais
2. **Au cours des 4 dernières semaines, quel a été votre niveau (degré) de désir sexuel ?**

- 5=Très élevé
- 4=Élevé
- 3=Moyen
- 2=Faible
- 1=Très faible ou inexistant

L'excitation sexuelle est une sensation qui comprend à la fois des aspects physiques et psychologiques. Elle peut comprendre des sensations de chaleur ou de picotement au niveau des organes génitaux, la lubrification (humidité) du vagin ou des contractions musculaires.

1. **Au cours des 4 dernières semaines, vous êtes-vous sentie excitée sexuellement pendant une activité sexuelle ou un rapport sexuel ?**

- 0=Aucune activité sexuelle
- 5=Presque toujours ou toujours
- 4=La plupart du temps (plus d'une fois sur deux)
- 3=Parfois (environ une fois sur deux)
- 2=Rarement (moins d'une fois sur deux)
- 1=Presque jamais ou jamais

1. **Au cours des 4 dernières semaines, quel a été votre niveau (degré) d'excitation sexuelle pendant une activité sexuelle ou un rapport sexuel ?**

- 0=Aucune activité sexuelle
- 5=Très élevé
- 4=Élevé
- 3=Moyen
- 2=Faible
- 1=Très faible ou inexistant

1. **Au cours des 4 dernières semaines, à quel point vous êtes-vous sentie sûre de votre capacité à être sexuellement excitée pendant une activité sexuelle ou un rapport sexuel ?**

- 0=Aucune activité sexuelle
- 5=Extrêmement sûre
- 4=Très sûre
- 3=Moyennement sûre
- 2=Peu sûre
- 1=Très peu sûre ou pas sûre du tout

1. **Au cours des 4 dernières semaines, avez-vous été satisfaite de votre degré d'excitation pendant une activité sexuelle ou un rapport sexuel ?**
   1. 0=Aucune activité sexuelle
   2. 5=Presque toujours ou toujours
   3. 4=La plupart du temps (plus d'une fois sur deux)
   4. 3=Parfois (environ une fois sur deux)
   5. 2=Rarement (moins d'une fois sur deux)
   6. 1=Presque jamais ou jamais
2. **Au cours des 4 dernières semaines, votre vagin était-il lubrifié (humide) pendant une activité sexuelle ou un rapport sexuel ?**
   1. 5=Presque toujours ou toujours
   2. 4=La plupart du temps (plus d'une fois sur deux)
   3. 3=Parfois (environ une fois sur deux)
   4. 2=Rarement (moins d'une fois sur deux)
   5. 1=Presque jamais ou jamais
   6. 0=Aucune activité sexuelle
3. **Au cours des 4 dernières semaines, à quel point vous a-t-il été difficile d'avoir le vagin lubrifié (humide) pendant une activité sexuelle ou un rapport sexuel ?**
   1. 0=Aucune activité sexuelle

- 1=Extrêmement difficile ou impossible
- 2=Très difficile
- 3=Difficile
- 4=Légèrement difficile
- 5=Pas difficile

1. **Au cours des 4 dernières semaines, la lubrification (humidité) de votre vagin a-t-elle duré jusqu'à la fin d'une activité sexuelle ou d'un rapport sexuel ?**
   1. 5=Presque toujours ou toujours
   2. 4=La plupart du temps (plus d'une fois sur deux)
   3. 3=Parfois (environ une fois sur deux)
   4. 2=Rarement (moins d'une fois sur deux)
   5. 1=Presque jamais ou jamais
   6. 0=Aucune activité sexuelle
2. **Au cours des 4 dernières semaines, à quel point vous a-t-il été difficile de conserver la lubrification (humidité) de votre vagin jusqu'à la fin d'une activité sexuelle ou d'un rapport sexuel ?**
   1. 0=Aucune activité sexuelle

- 1=Extrêmement difficile ou impossible
- 2=Très difficile
- 3=Difficile
- 4=Légèrement difficile
- 5=Pas difficile

1. **Au cours des 4 dernières semaines, lorsque vous avez été stimulée sexuellement ou que vous avez eu un rapport sexuel, avez-vous atteint l'orgasme ?**
   1. 5=Presque toujours ou toujours
   2. 4=La plupart du temps (plus d'une fois sur deux)
   3. 3=Parfois (environ une fois sur deux)
   4. 2=Rarement (moins d'une fois sur deux)
   5. 1=Presque jamais ou jamais
   6. 0=Aucune activité sexuelle
2. **Au cours des 4 dernières semaines, lorsque vous avez été stimulée sexuellement ou que vous avez eu un rapport sexuel, à quel point vous a-t-il été difficile d'atteindre l'orgasme ?**
   1. 0=Aucune activité sexuelle

- 1=Extrêmement difficile ou impossible
- 2=Très difficile
- 3=Difficile
- 4=Légèrement difficile
- 5=Pas difficile

1. **Au cours des 4 dernières semaines, à quel point avez-vous été satisfaite de votre capacité à atteindre l'orgasme pendant une activité sexuelle ou un rapport sexuel ?**

- 5=Très satisfaite
- 4=Moyennement satisfaite
- 3=Ni satisfaite, ni insatisfaite
- 2=Moyennement insatisfaite
- 1=Très insatisfaite
- 0=Aucune activité sexuelle

1. **Au cours des 4 dernières semaines, à quel point avez-vous été satisfaite de votre relation affective avec votre partenaire pendant une activité sexuelle ?**

- 5=Très satisfaite
- 4=Moyennement satisfaite
- 3=Ni satisfaite, ni insatisfaite
- 2=Moyennement insatisfaite
- 1=Très insatisfaite
- 0=Aucune activité sexuelle

1. **Au cours des 4 dernières semaines, à quel point avez-vous été satisfaite de votre relation avec votre partenaire du point de vue sexuel ?**

- 5=Très satisfaite
- 4=Moyennement satisfaite
- 3=Ni satisfaite, ni insatisfaite
- 2=Moyennement insatisfaite
- 1=Très insatisfaite

1. **Au cours des 4 dernières semaines, à quel point avez-vous été satisfaite de votre vie sexuelle en général ?**

- 5=Très satisfaite
- 4=Moyennement satisfaite
- 3=Ni satisfaite, ni insatisfaite
- 2=Moyennement insatisfaite
- 1=Très insatisfaite

1. **Au cours des 4 dernières semaines, avez-vous ressenti une gêne ou de la douleur pendant la pénétration vaginale ?**

- 0=Je n'ai pas eu de rapport sexuel
- 1=Presque toujours ou toujours
- 2=La plupart du temps (plus d'une fois sur deux)
- 3=Parfois (environ une fois sur deux)
- 4=Rarement (moins d'une fois sur deux)
- 5=Presque jamais ou jamais

1. **Au cours des 4 dernières semaines, avez-vous ressenti une gêne ou de la douleur après la pénétration vaginale ?**

- 0=Je n'ai pas eu de rapport sexuel
- 1=Presque toujours ou toujours
- 2=La plupart du temps (plus d'une fois sur deux)
- 3=Parfois (environ une fois sur deux)
- 4=Rarement (moins d'une fois sur deux)
- 5=Presque jamais ou jamais

1. **Au cours des 4 dernières semaines, quel a été votre niveau (degré) de gêne ou de douleur pendant ou après la pénétration vaginale ?**

- 0=Je n'ai pas eu de rapport sexuel
- 1=Très élevé
- 2=Élevé
- 3=Moyen
- 4=Faible
- 5=Très faible ou inexistant

**FGSIS**

1. **J’ai un sentiment positif concernant mes organes génitaux**

- 4= tout à fait d'accord
- 3= d'accord
- 2= pas d'accord
- 1= Pas du tout d'accord

1. **Je suis satisfait de l’aspect de mes organes génitaux**

- 4= tout à fait d'accord
- 3= d'accord
- 2= pas d'accord
- 1= Pas du tout d'accord

1. **Je serais à l’aise de laisse mon partenaire regardes mes organes génitaux**.

- 4= tout à fait d'accord
- 3= d'accord
- 2= pas d'accord
- 1= Pas du tout d'accord

1. **Je pense mes organes génitaux ont une bonne odeur**

- 4= tout à fait d'accord
- 3= d'accord
- 2= pas d'accord
- 1= Pas du tout d'accord

1. **Je pense mes organes génitaux fonctionne comme ils sont censés le faire**

- 4= tout à fait d'accord
- 3= d'accord
- 2= pas d'accord
- 1= Pas du tout d'accord

1. **Je suis à l’aise de laisser un professionnel de santé examiner mes organes génitaux**

- 4= tout à fait d'accord
- 3= d'accord
- 2= pas d'accord
- 1= Pas du tout d'accord

1. **Je ne suis pas gênée par mes organes génitaux**

- 4= tout à fait d'accord
- 3= d'accord
- 2= pas d'accord
- 1= Pas du tout d'accord

1. **Pensez-vous que les sept dernières questions nous ont donné une image réaliste de votre attitude envers vos organes génitaux ? (0=pas du tout réaliste, 10=tout à fait réaliste)**

- 0-10

1. **Etait-il stressant de remplir ce questionnaire ? (une seule réponse) (0=pas du tout stressant, 10= très stressant)**
   1. 0-10
2. **Y a-t-il quelque chose d’autre dont vous aimeriez nous faire part ? Une remarque, un sentiment ? (texte libre)**

*** uniquement pour les femmes qui ont vécu une reconstruction clitoridienne**

**** uniquement pour les femmes qui ont seulement vécu les soins psychosexuels**
