# Supplementary material for: Clitoral reconstruction and psychosexual care after female genital mutilation/cutting: Assessment of multidisciplinary care
Source: Womens Health (Lond). 2025 Mar 31;21:17455057251315814. doi: 10.1177/17455057251315814 (PMC11960189; doi:10.1177/17455057251315814)
Supplement: sj-docx-3-whe-10.1177_17455057251315814 – Supplemental material for Clitoral reconstruction and psychosexual care after female genital mutilation/cutting: Assessment of multidisciplinary care [file sj-docx-3-whe-10.1177_17455057251315814.docx]

Questionnaire

This is a multiple-choice questionnaire regarding the study: **Clitoral reconstruction and psychosexual care after FGM/C: Assessment of multidisciplinary care**

The interview is structured to last about 1 hour.

Your Sociodemographic Data

How old are you?

- 18-24 years
- 25-29 years
- 30-34 years
- 35-39 years
- 40-44 years
- 45-49 years
- >50 years

In which country were you born?

- Somalia
- Eritrea
- Ethiopia
- Sudan
- Mali
- Guinea
- Other: __________

Where is your mother from?

- Somalia
- Eritrea
- Ethiopia
- Sudan
- Mali
- Guinea
- Other: __________

Where is your father from?

- Somalia
- Eritrea
- Ethiopia
- Sudan
- Mali
- Guinea
- Other: __________

What languages do you speak? (Multiple answers possible)

- French
- English
- Tigrinya
- Somali
- Amharic
- Arabic
- Other: __________

Which language do you feel most comfortable in?
(Please enter your answer)

How long have you been living in Switzerland?

- <6 months
- 6 months – 1 year
- 1-2 years
- 2-5 years
- 5-10 years
- >10 years

What is your marital status?

- Single
- Married
- Divorced/separated
- Widowed
- In a partnership

How long have you been in your current relationship?

- <6 months
- 6 months – 1 year
- 1-2 years
- 2-5 years
- 5-10 years
- >10 years

Do you have children?

- Yes
- No

If yes, how many daughters?

- 0
- 1
- 2
- ≥3

If yes, how many sons?

- 0
- 1
- 2
- ≥3

Do you have children who were born in Switzerland?

- Yes
- No

What is your highest level of education?

- Never attended school
- Primary school
- Partial secondary school
- High school diploma
- Some university years
- University degree or higher qualification

What is your employment status? (Multiple answers possible)

Student

- I am learning French
- Employed
- Housewife
- Unemployed
- Retired
- Inactive/disabled
- Other: __________

Regarding Your Circumcision and FGM in General

Do you feel able to talk about it: (one answer per section)

With the medical staff?

- Yes
- No

With your partner?

- Yes
- No

With your loved ones?

- Yes
- No

How old were you when your excision was performed? (one answer only)

- Birth or a few days/months after
- <5 years
- 5-10 years
- 10-15 years
- 15-20 years
- >20 years
- I don’t know anymore

Why do you think your excision was performed? (Multiple answers possible)

- To become a woman
- To become pure
- To be respected
- To be able to marry one day
- To conform to the social and cultural norms of your community
- To be more beautiful
- To prevent hypersexuality
- To be cleaner
- To avoid judgment
- To be protected
- Other
- I don’t know anymore

How would you describe your experience at the time of the excision? (Multiple answers possible)

- Positive
- Negative
- Neutral
- Beautiful
- Ugly
- Painless
- Painful
- Other:
- I don’t remember

I felt: (Multiple answers possible)

- Brave
- Proud
- Honored
- Celebrated
- Betrayed
- Alone
- Angry
- Happy
- Unhappy
- Normal
- Abnormal
- Confident
- Adult
- Scared
- Other:
- I don’t remember

What do you feel now, having been excised? (Multiple answers possible)

- Respectable
- Proud
- Cleaner
- Free
- Angry
- Ashamed
- Disgusted
- Guilty
- Normal
- Different
- Abnormal
- Other:

Concerning your health before your "FGM" consultation appointment:

What symptoms did you have before the treatment? (Multiple answers possible)

- No symptoms
- Urinary (infections, flow obstruction, etc.)
- Genital pain outside of sexual intercourse
- Genital pain during sexual intercourse
- Psychological (fear, shame, guilt, trauma, etc.)
- Genital (cysts, recurrent itching, etc.)
- Sexual (lack of desire or pleasure, etc.)
- No sexual activity
- Other:

Do you think these symptoms were related to the excision? (One answer only)

- Yes
- No

Have you ever experienced sexual difficulties? (Multiple answers possible)

- No
- Yes. If yes, which ones:
- Bleeding during intercourse
- Psychological inability to have sexual intercourse
- Physical inability to have sexual intercourse
- Lack of pleasure during sexual intercourse
- Delayed/avoided sexual intercourse
- Pain during intercourse
- Lack of desire
- Injuries to the genital area during intercourse
- Lack of lubrication
- Inability to have an orgasm
- Other:

Before the multidisciplinary care, did you have a positive feeling about your genital organs? (One answer only)

- 4 = Strongly agree
- 3 = Agree
- 2 = Disagree
- 1 = Strongly disagree

Concerning your satisfaction with the care at the "FGM" consultation at HUG (psychosexual support and possibly clitoral reconstruction):

What did the multidisciplinary care consist of in your case? (One answer only)

- Psychosexual follow-up?
- How many consultations with a gynecologist?
- How many consultations with a sexologist/psychologist?
- Psychosexual follow-up and clitoral reconstruction

What did you expect/hope for from the surgical treatment? * (free text)

Were your expectations satisfied by the surgical therapy? * (One answer only)

- Yes, totally
- Yes, partially
- No, not at all
- Why? (free text)

Were your expectations satisfied by the psychosexual therapy? (One answer only)

- Yes, totally
- Yes, partially
- No, not at all
- Why? (free text)

How satisfied are you with the multidisciplinary therapy on a scale of 0 to 10 (0 = not satisfied at all, 10 = fully satisfied)? (One answer only)

Do you think that the psychosexual care led to an overall improvement: (One answer per section) (0 = strongly disagree, 10 = strongly agree)

- In your health? (0-10)
- In your sexuality? (0-10)
- In your genital image? (0-10)
- In genital pain during intercourse? (0-10)
- In genital pain outside of intercourse? (0-10)

Do you think that the clitoral reconstruction led to an overall improvement: * (One answer per section) (0 = strongly disagree, 10 = strongly agree)

- In your health? (0-10)
- In your sexuality? (0-10)
- In your genital image? (0-10)
- In genital pain during intercourse? (0-10)
- In genital pain outside of intercourse? (0-10)

After clitoral reconstruction, how do you see yourself? (Multiple answers possible) *

- Respectable
- Proud
- Clean
- Free
- Sexy
- Normal
- Beautiful
- Complete
- Angry
- Ashamed
- Disgusted
- Guilty
- With regrets
- I don’t notice any change
- Other:

Did you have any fears about the surgery? * (One answer only)

- Yes
- No

If yes: What were they? Were they justified? (free text)

Would you recommend your follow-up to other excised women? (One answer only)

- Yes
- No

Do you think psychosexual care is essential for excised women? (One answer only)

- Yes
- No

Do you think clitoral reconstruction is essential for excised women? (One answer only)

- Yes
- No

Why did you ultimately decide not to undergo clitoral reconstruction? (Multiple answers possible) **

- Other expectations regarding clitoral reconstruction
- Needs met through psychosexual care
- Medical contraindications
- Social circumstances
- Breaking the taboo
- Fear of the surgery
- Uncertainty about the result
- Feeling misunderstood
- Need for deeper reflection
- I had surgery elsewhere
- Other priorities
- Treatment process was too long
- Fear of pain
- Fear that my sexuality might worsen
- I haven’t given up, I’m just waiting for the right moment
- Other

What helped you the most personally? (Multiple answers possible)

- Talking
- Having a contact person
- Education on the function and anatomy of female genital organs
- Psychological support
- Surgical treatment
- Other

Were there things that you feel were missing in or during your care? (free text)

Does your partner know about the consultations at HUG? (One answer only)

- Yes
- No

If yes, what is their attitude towards the consultations? (Multiple answers possible):

- Positive
- Negative
- Proud
- Mean
- Sad
- Other:

Concerning your motivation to consult at HUG:

What was your motivation for clitoral reconstruction? (Multiple answers possible)

- Genital pain during intercourse
- Genital pain outside of intercourse
- Reconstruction of my identity
- To obtain a repair
- Improve my body/genital image
- Improve my sexuality
- To feel like a complete woman (normality)
- To be like other women (equality)
- To recover what was taken from me (justice)
- Other:

Was there a main motivation for you? (One answer only)

- Yes
- No

If yes, which one? (free text)

How did you discover multidisciplinary care / clitoral reconstruction at HUG? (Multiple answers possible)

- From friends
- From a doctor (gynecologist, family doctor, etc.)
- From my partner
- On the internet
- On social media
- Other:
- How long did you think about consulting at HUG before making an appointment?

Concerning your current health:

How would you describe your mental health? (Multiple answers possible)

- Good
- Poor
- Known mental health disorder, namely:
- What symptoms are you currently experiencing? (Multiple answers possible)
- Urinary (infections, flow obstruction, etc.)
- Genital pain during intercourse
- Genital pain outside of intercourse
- Psychological (fear, shame, guilt, trauma, etc.)
- Genital (cysts, recurrent itching, etc.)
- Sexual (pain, lack of pleasure, etc.)
- No sexuality
- No symptoms
- Other:

Are they linked to:

The excision? (One answer only)

- Yes
- No

The surgery? (One answer only)

- Yes
- No

Do you currently have sexual difficulties? (Multiple answers possible)

- No
- Yes. If yes, which ones:
- Bleeding during intercourse
- Psychological inability to have sexual intercourse
- Physical inability to have sexual intercourse
- Lack of pleasure during sexual intercourse
- Delayed / postponed sexual intercourse
- Pain during intercourse
- Lack of desire
- Injuries to genital areas during intercourse
- Lack of lubrication
- Inability to reach orgasm
- Other:

Do you have a visible clitoris since the clitoral reconstruction? (One answer only)

- Yes
- No

Well-being: WHO Five Well-being Index (1999)

Over the past two weeks:

I felt good and in a good mood:

- 5 = All the time
- 4 = Most of the time
- 3 = More than half of the time
- 2 = Less than half of the time
- 1 = Occasionally
- 0 = Never

I felt calm and relaxed:

- 5 = All the time
- 4 = Most of the time
- 3 = More than half of the time
- 2 = Less than half of the time
- 1 = Occasionally
- 0 = Never

I felt full of energy and vigor:

- 5 = All the time
- 4 = Most of the time
- 3 = More than half of the time
- 2 = Less than half of the time
- 1 = Occasionally
- 0 = Never

I woke up feeling fresh and rested:

- 5 = All the time
- 4 = Most of the time
- 3 = More than half of the time
- 2 = Less than half of the time
- 1 = Occasionally
- 0 = Never

My daily life has been filled with interesting things:

- 5 = All the time
- 4 = Most of the time
- 3 = More than half of the time
- 2 = Less than half of the time
- 1 = Occasionally
- 0 = Never

FSFI (Female Sexual Function Index)

The following questions concern your feelings and reactions about your sexual activity in the last 4 weeks. Please answer these questions as sincerely and clearly as possible. Your answers will remain strictly confidential. When responding, please consider the following definitions:

Sexual activity can include caresses, foreplay, masturbation, and vaginal penetration.

Sexual intercourse is defined as penile penetration (insertion).

Sexual arousal includes physical and psychological aspects, such as sensations of heat or tingling in the genital area, vaginal lubrication (moisture), or muscle contractions.

Sexual desire is the feeling of wanting to engage in sexual activity, being receptive to a partner's advances, and having sexual thoughts or fantasies.

Over the past 4 weeks, did you feel sexual desire?

- 5 = Almost always or always
- 4 = Most of the time (more than half the time)
- 3 = Sometimes (about half the time)
- 2 = Rarely (less than half the time)
- 1 = Almost never or never

Over the past 4 weeks, how would you rate your level of sexual desire?

- 5 = Very high
- 4 = High
- 3 = Medium
- 2 = Low
- 1 = Very low or nonexistent

Over the past 4 weeks, did you feel sexually aroused during sexual activity or intercourse?

- 0 = No sexual activity
- 5 = Almost always or always
- 4 = Most of the time (more than half the time)
- 3 = Sometimes (about half the time)
- 2 = Rarely (less than half the time)
- 1 = Almost never or never

Over the past 4 weeks, how would you rate your level of sexual arousal during sexual activity or intercourse?

- 0 = No sexual activity
- 5 = Very high
- 4 = High
- 3 = Medium
- 2 = Low
- 1 = Very low or nonexistent

Over the past 4 weeks, how confident did you feel in your ability to become sexually aroused during sexual activity or intercourse?

- 0 = No sexual activity
- 5 = Extremely confident
- 4 = Very confident
- 3 = Moderately confident
- 2 = Slightly confident
- 1 = Very little or not confident at all

Over the past 4 weeks, were you satisfied with your level of sexual arousal during sexual activity or intercourse?

- 0 = No sexual activity
- 5 = Almost always or always
- 4 = Most of the time (more than half the time)
- 3 = Sometimes (about half the time)
- 2 = Rarely (less than half the time)
- 1 = Almost never or never

Over the past 4 weeks, was your vagina lubricated (moist) during sexual activity or intercourse?

- 5 = Almost always or always
- 4 = Most of the time (more than half the time)
- 3 = Sometimes (about half the time)
- 2 = Rarely (less than half the time)
- 1 = Almost never or never
- 0 = No sexual activity

Over the past 4 weeks, how difficult was it for you to become lubricated (moist) during sexual activity or intercourse?

- 0 = No sexual activity
- 1 = Extremely difficult or impossible
- 2 = Very difficult
- 3 = Difficult
- 4 = Slightly difficult
- 5 = Not difficult

Over the past 4 weeks, did the lubrication (moisture) of your vagina last until the end of a sexual activity or intercourse?

- 5 = Almost always or always
- 4 = Most of the time (more than half the time)
- 3 = Sometimes (about half the time)
- 2 = Rarely (less than half the time)
- 1 = Almost never or never
- 0 = No sexual activity

Over the past 4 weeks, how difficult was it for you to maintain vaginal lubrication (moisture) until the end of a sexual activity or intercourse?

- 0 = No sexual activity
- 1 = Extremely difficult or impossible
- 2 = Very difficult
- 3 = Difficult
- 4 = Slightly difficult
- 5 = Not difficult

Over the past 4 weeks, when you were sexually stimulated or had intercourse, did you reach orgasm?

- 5 = Almost always or always
- 4 = Most of the time (more than half the time)
- 3 = Sometimes (about half the time)
- 2 = Rarely (less than half the time)
- 1 = Almost never or never
- 0 = No sexual activity

Over the past 4 weeks, how difficult was it for you to reach orgasm during sexual activity or intercourse?

- 0 = No sexual activity
- 1 = Extremely difficult or impossible
- 2 = Very difficult
- 3 = Difficult
- 4 = Slightly difficult
- 5 = Not difficult

Over the past 4 weeks, how satisfied were you with your ability to reach orgasm during sexual activity or intercourse?

- 5 = Very satisfied
- 4 = Moderately satisfied
- 3 = Neither satisfied nor dissatisfied
- 2 = Moderately dissatisfied
- 1 = Very dissatisfied
- 0 = No sexual activity

Over the past 4 weeks, how satisfied were you with your emotional connection with your partner during sexual activity?

- 5 = Very satisfied
- 4 = Moderately satisfied
- 3 = Neither satisfied nor dissatisfied
- 2 = Moderately dissatisfied
- 1 = Very dissatisfied
- 0 = No sexual activity

Over the past 4 weeks, how satisfied were you with your sexual relationship with your partner?

- 5 = Very satisfied
- 4 = Moderately satisfied
- 3 = Neither satisfied nor dissatisfied
- 2 = Moderately dissatisfied
- 1 = Very dissatisfied

Over the past 4 weeks, how satisfied were you with your sex life in general?

- 5 = Very satisfied
- 4 = Moderately satisfied
- 3 = Neither satisfied nor dissatisfied
- 2 = Moderately dissatisfied
- 1 = Very dissatisfied

Over the past 4 weeks, did you experience discomfort or pain during vaginal penetration?

- 0 = No sexual activity
- 1 = Almost always or always
- 2 = Most of the time (more than half the time)
- 3 = Sometimes (about half the time)
- 4 = Rarely (less than half the time)
- 5 = Almost never or never

Over the past 4 weeks, did you experience discomfort or pain after vaginal penetration?

- 0 = No sexual activity
- 1 = Almost always or always
- 2 = Most of the time (more than half the time)
- 3 = Sometimes (about half the time)
- 4 = Rarely (less than half the time)
- 5 = Almost never or never

Over the past 4 weeks, how severe was the discomfort or pain during or after vaginal penetration?

- 0 = No sexual activity
- 1 = Very high
- 2 = High
- 3 = Medium
- 4 = Low
- 5 = Very low or nonexistent

FGSIS (Female Genital Self-Image Scale)

I have a positive feeling about my genitals.

- 4 = Strongly agree
- 3 = Agree
- 2 = Disagree
- 1 = Strongly disagree

I am satisfied with the appearance of my genitals.

- 4 = Strongly agree
- 3 = Agree
- 2 = Disagree
- 1 = Strongly disagree

I would be comfortable letting my partner see my genitals.

- 4 = Strongly agree
- 3 = Agree
- 2 = Disagree
- 1 = Strongly disagree

I think my genitals have a good smell.

- 4 = Strongly agree
- 3 = Agree
- 2 = Disagree
- 1 = Strongly disagree

I think my genitals function as they are supposed to.

- 4 = Strongly agree
- 3 = Agree
- 2 = Disagree
- 1 = Strongly disagree

I am comfortable with a healthcare professional examining my genitals.

- 4 = Strongly agree
- 3 = Agree
- 2 = Disagree
- 1 = Strongly disagree

I am not embarrassed by my genitals.

- 4 = Strongly agree
- 3 = Agree
- 2 = Disagree
- 1 = Strongly disagree

Do you think the last seven questions gave us a realistic picture of your attitude towards your genitals? (0 = not realistic at all, 10 = totally realistic)

0–10

Was it stressful to fill out this questionnaire? (One answer only) (0 = not at all stressful, 10 = very stressful)

0–10

Is there anything else you would like to share with us? A comment or feeling? (free text)

* only for women who have undergone clitoral reconstruction

** only for women who have undergone psychosexual care only
